# Evaluating a targeted Palbociclib-Trastuzumab loaded smart niosome platform for treating HER2 positive breast cancer cells

**DOI:** 10.1016/j.ijpx.2024.100237

**Published:** 2024-03-11

**Authors:** Shaghayegh Saharkhiz, Negar Nasri, Nazanin Naderi, Ghasem Dini, Saeid Shirzadi Ghalehshahi, Fateme Firoozbakht

**Affiliations:** a Department of Biotechnology, Faculty of Biological Science and Technology, University of Isfahan, Isfahan 81746-73441, Iran; b Department of Cell and Molecular Biology, Faculty of Life Science and Biotechnology, Shahid Beheshti University, Tehran 19839-69411, Iran; c Department of Nanotechnology, Faculty of Chemistry, University of Isfahan, Isfahan 81746-73441, Iran

**Keywords:** Drug delivery, Trastuzumab, Monoclonal antibody, pH-sensitive, Niosome

## Abstract

In this study, we present a targeted and pH-sensitive niosomal (pHSN) formulation, incorporating quantum dot (QD)-labeled Trastuzumab (Trz) molecules for the specific delivery of Palbociclib (Pal) to cells overexpressing human epidermal growth factor receptor 2 (HER2). FTIR analyses confirmed the successful preparation of the pHSNs and their bioconjugation. The labeled Trz-conjugated Pal-pHSNs (Trz-Pal-pHSNs) exhibited a size of approximately 170 nm, displaying a spherical shape with a neutral surface charge of −1.2 mV. Pal encapsulation reached ∼86%, and the release pattern followed a two-phase pH-dependent mechanism. MTT assessments demonstrated enhanced apoptosis induction, particularly in HER2-positive cells, by Trz-Pal-pHSNs. Fluorescence imaging further validated the internalization of particles into cells. In conclusion, Trz-Pal-pHSNs emerge as a promising platform for personalized medicine in the treatment of HER2-positive breast cancer.

## Graphical abstract

**Figure:**
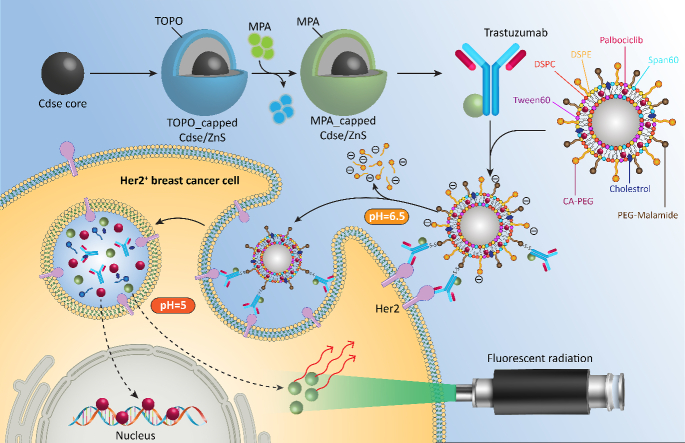

## 1 Introduction

Breast cancer (BC) is one of the most common cancers among women worldwide (Tsilidis et al., 2023). Three types of BC can be distinguished based on the presence or absence of specific proteins. Seventy percent of BCs are hormone receptor-positive, containing either estrogen receptors (ER) or progesterone receptors (PR). Fifteen to 20% of BC cases are ERBB2-positive (formerly known as HER2-positive), characterized by cancer cells containing high levels of the ERBB2 protein. Additionally, 15% of BC cases are triple-negative, lacking ER, PR, or ERBB2 proteins. Amplification of the HER2 gene or overexpression of the HER2 receptor are hallmarks of HER2-positive BCs, driving the selection of HER2-directed therapies (Murthy et al., 2014). As one of the most effective treatments for metastatic BC, Trastuzumab (Trz) has been added to standard chemotherapy regimens since the late 1990s and has demonstrated an improvement in both disease-free survival and overall survival (Lin and Rugo, 2007). In the majority of patients with HER2-positive metastatic BC, Trz secondary resistance develops within a year, and more than one-third fail to respond to the medication (Choong et al., 2020).

The efficacy of targeting cyclin-dependent kinases (CDKs), such as CDK4/6, has been proposed for women with HER2+ BC. CDKs play a crucial role in regulating cell-cycle transitions, specifically overseeing the transition from the G1 to the S phase. Many tumors, including BC exhibit dysregulation of the CDK4/6-cyclin D—Rb pathway, prompting the development of CDK4/6 inhibitors that induce G1 arrest and apoptosis. BC cyclins respond to growth stimuli, such as receptor tyrosine kinases, estrogen receptors, and progesterone receptors. In cell models, blocking CDK4/6 effectively inhibited growth (Rocca et al., 2014). Palbociclib (Pal), a new-generation drug prescribed for patients with HER2-positive cancer, halts the cell cycle and induces apoptosis by inhibiting CDK4/6. Studies demonstrate that Pal is most effective in luminal-type ER-positive cancer cells, including those amplified by HER2, and it synergizes with Trz in luminal-type ER-positive cancer cells (Ciruelos et al., 2020). Throughout the treatment course, the combination of Trz antibody and Pal exhibited a synergistic effect, significantly improving disease progression. However, this combination led to occurrences of neutropenia and thrombocytopenia. Currently, there are no satisfactory results from the conventional methods of endocrine or immunotherapy and chemotherapy for treating HER2-positive cancer due to their severe side effects (Gampenrieder et al., 2020). Therefore, using the smart nanocarriers can help to reduce the side effects of both Pal and Trz in addition to benefiting from the advantages of their combinational administration (Rana et al., 2023; Ademuyiwa et al., 2023a). This strategy of designing smart nanocarriers that respond to the unique environmental features of the cancerous cells can enhance the precision of the treatment specifically to the cancerous cells besides reducing their effect on normal cells (Wang et al., n.d.).

A major drawback of chemotherapy is the low bioavailability of high molecular weight chemotherapeutic agents. To overcome this limitation, a more effective site-specific drug delivery system (DDS) is needed to enhance the efficacy of therapeutic agents with minimal toxicity for healthy cells. To advance research toward this goal, several nanocarriers have been explored in the treatment of BC. Among these, vesicular nanocarriers such as Niosomes are particularly intriguing due to their unique characteristics, including the ability to transport hydrophilic and hydrophobic drugs, high biocompatibility, improved pharmacokinetic properties, enhanced solubility of chemotherapy drugs, and optimal drug release (Di Francesco et al., 2021). In detail, Niosomes are self-association vesicles composed of non-ionic surfactants and other additives such as cholesterol and phospholipids, used for drug delivery purposes (Gharbavi et al., 2018). The selectivity of these nanocarriers can be further enhanced using three strategies: active targeting, passive targeting, and smart targeting (Attia et al., 2019). Through passive transmission, these systems maintain enhanced permeability and retention (EPR) into tumor cells at higher concentrations due to leaky vasculature in cancer tissue endothelium (Escrivá-de-Romaní et al., 2018). In the treatment of HER2-positive BC, targeting the overexpression of HER2 antigen on the surface of cancer cells is a potent strategy for the formulation of targeted nanocarriers. Drug nanocarriers can be functionalized with HER2 antibodies for active transfer (Sadat et al., 2015). In addition, Smart targeting involves delivering a drug tailored to the specific characteristics of the tissue, such as the pH and temperature difference between tumor cells and healthy cells. Previously, our team introduced a new type of polymer (1, 2-Distearoyl-sn-glycero-3-phosphoethanolamine-Citraconic anhydride-Poly ethylene glycol (DSPE-CA-PEG)), which works based on changing the pH in a way that the structure of the smart polymer, sensitive to pH, forms a reversible bond with citraconic anhydride. This bond, in the presence of an amine agent, results in the formation of citraconic-amide. In a neutral state, it carries a negative charge, but in acidic conditions (around cancer cells), its charge changes to positive. Incorporating of this polymer in the composition of a niosomal nanoparticle provide pH-sensitivity of the niosomes, causing the collapse of the niosome structure in acidic environment of cancer tissue, while is stable in the neutral pH of normal tissues (Gadag et al., 2020).

**Scheme 1:**
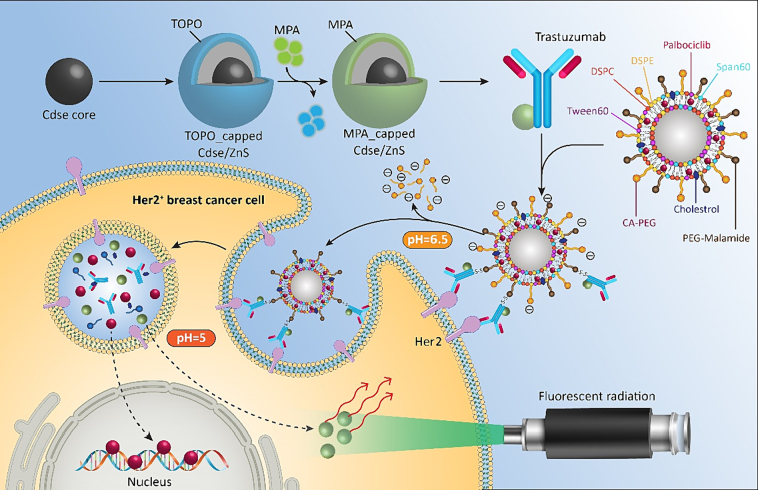
The overall strategy, fabrication, and assessment methods used in this study to develop a targeted Pal-Trz loaded smart niosome platform for treating HER2 positive BC cells.

In this study (Scheme 1), we employed a niosomal-targeted theranostic system containing a new-generation drug (Pal) effective on the cell cycle. In detail, to create pH sensitivity in the niosomal formulation, pH-responsive citraconic-amide bonds were formed in the DSPE-CA-PEG2000 components of the niosomes. These bonds help to add the selectivity of the niosomes to the target cells in addition to the other advantages of niosomal particles including biodegradability, low toxicity, biocompatibility, ease of storage, etc. CdSe/ZnS quantum dots (QDs) were utilized to track and confirm the targeting of the system. The synergistic effect of Pal, together with Trz as a monoclonal antibody (mAb), was investigated on both HER2-positive BC cells and healthy cells. The study initiated the synthesis and modification of CdSe/ZnS QDs, subsequently attached to an anti-HER2 antibody via an amide bond. For targeted delivery of Pal to HER2-positive BC cells, the QD-labeled mAb was bio-conjugated to a pHSN system. The fabricated theranostic platforms underwent testing on both HER2-positive SKBR3 and normal L929 cells. Various characterization techniques were employed, including measuring the encapsulation percentage, investigating the release profile, and determining the bioactivity of the particles using an MTT test. The smart system's ability to induce apoptosis in HER2-positive cells was measured using an apoptosis kit and flow cytometry. Additionally, the smart system, utilizing CdSe/ZnS QDs, confirmed the success of the system and tracked its performance. The fluorescent effect of the QDs was evaluated once the system was taken up, and the drug was released.

## 2 Materials and methods

### 2.1 Materials

1,2-distearoyl-sn-glycero-3-phosphoethanolamine-N-[maleimide(polyethylene glycol)-2000] (ammonium salt) (DSPE-PEG (2000)-Maleimide, ≥99), Polyoxyethylene sorbitan monolaurate (Tween 20, ≥99%), 3β-Hydroxy-5-cholestene (Chol, ≥99%), Sorbitan stearate (Span 60, ≥99%), ethanol-1,1,2,2-d4-amine (≥99%), Albumin bovine serum (BSA, ≥98%), 2-thiolanimine hydrochloride (≥99%), Roswell Park Memorial Institute 1640 medium (RPMI-1640) including 10% Fetuin (FBS), Thiazolyl blue formazan (MTT) powder, sodium tetraborate (Borax, ≥98%), boric acid (≥99.5%), Drabkin's reagent and dimethylsulfoxide (DMSO) were purchased from Sigma-Aldrich, Darmstadt, Germany. Chloroform, 1-(3-dimethyl aminopropyl)-3-ethyl carbodiimide hydrochloride (EDC), N-hydroxysuccinimide (NHS) and mercaptopropionic acid (MPA, ≥99%) and diethyl ether (≥ 99.9%), 2-propanol and ethanol were bought from Merck, Darmstadt, Germany. Trastuzumab monoclonal antibody was purchased from Roche Company, Branchburg, New Jersey, USA. In addition, Trypsin / EDTA: 0.025% Trypsin / 0.01% EDTA and penicillin/streptomycin antibiotics were acquired from Thermofisher, Berman, Germany. Palbociclib (≥99%) was purchased from Actore Co, Karaj Iran. The chemicals were used without further purification. The SKBR-3 and L929 cell lines also were acquired from the Pasteur Institute of Iran, Tehran, Iran.

### 2.2 Methods

#### 2.2.1 Synthesis of drug-loaded pH-responsive niosome

In this study, we employed the thin-film hydration method to fabricate pHSNs loaded with the drug. Previously, our team had prepared DSPE-CA-PEG2000 for pH-sensitive purposes, and DSPE-PEG2000-Mal, DSPC, Tween 60, Span 60, and Chol were used for the niosomal components (Saharkhiz et al., 2023a). To dissolve all components, 5 mL of chloroform was used, and the mixture was stirred for 15 min in a molar ratio of 5:1.5:3.5:15:45:30. Subsequently, Pal was added to the solution, and it was vaporized at 60 °C under a vacuum condition to form a uniform thin film. In the next step, 10 mL of pre-heated PBS (50 mM, pH = 7.4) buffer solution was added to the thin film and stirred for 1 h at 60 °C to produce multiple-lamellar niosomes. Using a probe sonicator, single-lamellar niosomes were formed by sonicating the solution in an ice bath for 1 h. Finally, for purification, a dialysis method (12,000 Da cut-off, against PBS) was employed (Zhang, 2017).

#### 2.2.2 Ligand exchange of CdSe/ZnS QDs

The fabrication of TOPO-capped CdSe/ZnS QDs followed our previously established methods (Saharkhiz et al., 2023a; Saharkhiz et al., 2023b). Subsequently, a PBS solution was employed to disperse CdSe/ZnS QDs capped with TOPO. The next step involved replacing TOPO with mercaptopropionic acid (MPA). The carboxylate ligands present in MPA molecules contribute to the water dispersibility of QDs. To achieve this, a solution containing 1 mL of CdSe/ZnS QDs was stirred overnight with 5 μL of MPA and 1 mL of PBS (pH = 7.4, 50 mM). Following the reaction, the QDs were purified by centrifugation several times at 10,000 rpm for 15 min to remove any residual MPA (Saharkhiz et al., 2023b; Vo et al., 2015).

#### 2.2.3 Modification of QDs and bioconjugation with Trz antibody

In the initial step, MPA-CdSe/ZnS QDs were diluted 200 times in water. A reaction mixture containing 1-(3-dimethylaminopropyl)-3-ethyl carbodiimide hydrochloride (EDC), N-hydroxysuccinimide (NHS), and QDs was prepared in water without exposure to light for 2 h at a molar ratio of 1000:1000:1. To eliminate any remaining NHS and EDC in the solvent, functionalized QDs underwent ultrafiltration (30 kDa MW cut-off) (Wu et al., 2015). The CdSe/ZnS QDs with sulfo-NHS termination were then reconstituted in PBS at pH 7.4 and 4 °C. Subsequently, 1 mg/mL of Trz antibody was added, and the mixtures were incubated at 4 °C for 24 h with agitation. After the conjugation of CdSe/ZnS QDs with the antibody was complete, any remaining active functional groups were blocked with 0.1 M (3 μL) ethanolamine in 1 mL PBS. The biofunctionalized particles were washed ten times with PBS, and after centrifugation at 10,000 rpm at 4 °C for 15 min, Trz-QDs were purified by ultrafiltration and dispersed in PBS (Hua et al., 2006). In the final step, the QDs-Trz antibodies were conjugated to the maleimide groups of DSPE-PEG-mal components using the previously reported procedure by our team (Dvorakova et al., 2017).

### 2.3 Characterization

Zeta potential and dynamic light scattering (DLS) were employed to measure the size and surface charge of the QDs using a HORIBA Scientific SZ-100 instrument. Additionally, the exact size of the QDs was determined through transmission electron microscopy (TEM) using the HT7800 model from Hitachi. CdSe/ZnS core/shell QDs underwent evaluation using X-ray diffraction with a Bruker D8 Advance instrument. The construction of both QDs and MPA-QDs was assessed using Fourier-transform infrared spectroscopy (FTIR) with a JASCO 6300 instrument. Furthermore, fluorescence spectroscopy was utilized before and after the modifications to estimate the QDs' emission and excitation wavelengths.

DLS and zeta potential analyses were employed to compare the size and surface charge of pH-responsive niosomes before and after drug encapsulation. Additionally, FTIR analysis was conducted to verify the correct construction of empty niosomes, their encapsulation with the drug, and the subsequent conjugation with the Trz antibody. Scanning electron microscopy (SEM) using a Leo 1430 VP instrument was utilized to examine the morphology of niosomes both before and after modification. Furthermore, Nano ZS, a product of Malvern Instruments, was used to measure the zeta potential and size of the mAb-QDs-niosome.

### 2.4 Bioactivity assessments

#### 2.4.1 In vitro entrapment efficiency and loading capacity assessments

To determine the amount of drug present in nanoparticles, 300 μL of Pal-loaded niosomal suspension was mixed with 2700 μL of 2-propanol (1% *w*/*v* in water) and stirred at room temperature for approximately 48 h. After centrifugation at 10,000 rpm, the supernatant was analyzed using a UV spectrophotometer (Systronics, India) at a wavelength of 417 nm. All measurements were performed in triplicate. To determine the Pal concentration, a calibration curve was generated using a standard Pal solution. The curve exhibited a linear relationship between 5 and 40 μg/mL with a correlation coefficient of 0.9998. Using eqs. (1), (2), the entrapment efficiency percentage (EE%) and loading capacity (LC%) could be calculated (Shin et al., 2020):

(1) $$\text{Entrapment Efficiency}\;\left(\%\right)=\frac{\text{Mass}\;\left(\text{total drug}–\text{unloaded drug}\right)\;}{\;\text{Mass}\;\left(\text{total drug}\right)\;}\times 100$$

(2) $$\text{Loading Capacity}\;\left(\%\right)=\frac{\text{Mass}\;\left(\text{total drug}–\text{unloaded drug}\right)\;}{\;\text{Mass}\;\left(\text{total targeted nanoparticles}\right)\;}\times 100$$

#### 2.4.2 Pal release pattern and kinetic modeling

To assess the release pattern of Pal from the fabricated pHSNs, 1 mg of the final sample was placed in a dialysis bag (12 kDa MW cut-off), then dialyzed against 4 mL PBS solution with two different pH (5 and 7.4) at 37 °C. In the next step, 1 mL of the external media was collected at defined times (1, 2, 4, 6, 12, 24, 48, 72, and 120 h), then the absorbance of the collected samples was measured using a UV–Visible spectrometer device at a wavelength of 220 nm. In addition, the mechanism of Pal release from the prepared pHSNs was determined by employing equations that are described in Table 1 (Zhang et al., 2010; Saharkhiz et al., 2023c):

**Table 1:** Equations of Drug release kinetic models.

Number	Model of Release kinetic	Equation⁎
1	Zero-order	C = k_0_t
2	First-order	LogC_0_ – LogC_t_ = kt/ 2.303
3	Higuchi	Log Q = log k_H_ + 1/2log t
4	Hixson-Crowell	Q_0_^1/3^ – Q_t_^1/3^ = k_HCt_

⁎ 1) C = drug concentration, k_0_ = rate constant of the zero-order model, t = time, 2) C_0_ = the drugs initial concentration, C_t_ = drug released amount in time t, 3). Q = drug released amount in time t per unit area, k_H_ = Higuchi dissolution constant, and 4) Q_0_ = drug initial amount, Q_t_ = remained drug amount at time t, k_HCt_ = Hixson-Crowell rate constant (Zhang et al., 2010; Chime et al., 2013).

#### 2.4.3 Number of Trz attached to niosome

To quantify the number of monoclonal antibodies (mAbs) conjugated to each niosome, the Bradford protein assay was employed, following a previously published procedure by our group (Shin et al., 2020; Shin et al., 2016).

#### 2.4.4 Stability evaluation

After four months of storage at 4 °C, DLS and SEM analyses were repeated to assess any changes in size and EE% of the prepared samples.

#### 2.4.5 Hemolysis assay

An assay for hemolysis was conducted according to the standard method (Zhen et al., 2015). A 5-min centrifugation at 600 *g* was performed on each blood sample aliquot. 225 μL of Drabkin's reagent was added to a 25-μl plasma aliquot in a 96-well plate and mixed for two minutes under lateral agitation (300 rpm). Following 10 min of room temperature equilibration, optical density at 540 nm was measured using an Anthos HTIII multi-plate reader (Anthos Mikrosysteme GmbH, Germany). In order to determine whole blood hemoglobin, a 100-fold dilution was analyzed at 540 nm in Drabkin's reagent. A positive control was saponin (2 mg/ml final blood concentration) and a negative control was PBS. Baseline conditions refer to plasma without additives. A calibration curve was obtained using standard solutions containing 0.072 to 3.6 mg/ml bovine hemoglobin (Sigma) treated with Drabkin's reagent. Hemolysis is expressed as a percentage, i. e. free plasma hemoglobin (mg/ml) released as a result of contact with the test material divided by the total blood hemoglobin (mg/ml) multiplied by 100 (Kuznetsova et al., 2012).

#### 2.4.6 Prothrombin time (PT) and activated partial thromboplastin time (*a*PTT)

The aliquots of each blood sample were centrifuged for 5 min at 2000 *g*. The clotting times of plasma samples were measured on a Behring Coagulation Timer (Dade Behring Holdings Inc., US) with commercial reagents (Thromborel® S, Dade Behring/Siemens, for PT and C.K. PREST kit, Roche Diagnostics, France, for *a*PTT). Blood coagulation factors, including prothrombin time and activated partial thromboplastin time, were measured using an automation blood coagulation timer in comparison to platelet-poor plasma without nanoparticles. As a way to verify that the surface of the niosomes under study was not triggering the coagulation cascade. A human plasma standard (Dade Behring/Siemens) was used to calibrate the equipment (Cheraghali et al., 2023; Faraji et al., 2019).

#### 2.4.7 MTT assay

An MTT assay was employed to assess the cytotoxicity of niosomes, both with and without surface modifications, on SKBR-3 and L929 cells. SK-BR-3 is a human BC cell line that overexpresses the HER2 (approximately 10^6^ HER2 per cell) (Pasteur Institute, Tehran, Iran) (Ekerljung et al., 2008). The SKBR-3 cells were cultured in RPMI-1640 medium and it was modified to contain 2 mM l-glutamine, fetal bovine serum to a final concentration of 10%, 1% penicillin/streptomycin, and 1% non-essential amino acids. L-929 is an adherent type of mouse fibroblast cell line that was used as a negative control. DMEM/H+ medium with 2 mM l-glutamine and fetal bovine serum to a final concentration of 10%, 1% penicillin/streptomycin, and 1% non-essential amino acids was used for culturing of the L929 cell line. The cells were seeded in 96-well plates at a density of 10 × 10^3^ cells per well and incubated for 24 h in RPMI or DMEM medium at 37 °C with 5% CO2. Subsequently, 200 μL of freshly prepared RPMI or DMEM medium, containing 50 or 250 μg/mL of empty niosomes and drug-loaded niosomes, respectively, were added to each well. Similar concentrations of pure Pal and Trz were also used to treat the cells. The cells were then incubated for 24 and 48 h at 37 °C. After two washes with PBS (pH = 7.3), 200 μL of culture medium containing MTT solution (5 mg/mL in PBS) was added to each well, followed by incubation at 37 °C for 2 h. Once the MTT culture medium was removed, 100 μL of dimethyl sulfoxide (DMSO) was added to each well, and the absorbance was determined using a 570 nm ELISA reader (Bio-Rad, Hercules, CA, USA) (Saharkhiz et al., 2023b; Saharkhiz et al., 2023d).

#### 2.4.8 Apoptosis investigation

To assess the extent of apoptosis, flow cytometry was employed. SKBR-3 cells were seeded in 10 mm dishes and cultured until reaching 70% confluence. Each dish was then treated with 1 mL of fresh medium containing empty niosomes, drug-loaded niosomes, Trz-niosomes, and pure Pal. Following an overnight treatment, the cells were stained with Annexin V (5 μL) and propidium iodide (PI) (5 μL) dyes and incubated in a dark room for 15 min. Subsequently, the cells were analyzed using a flow cytometer (C6, BD Accuri, NJ, USA) (Saharkhiz et al., 2023a).

#### 2.4.9 Bio-imaging

To assess and validate the theranostic capability of the system, a fluorescence microscope was employed. Following a 24-h incubation of SKBR-3 and L929 cells with mAb-conjugated QDs loaded niosomes at 37 °C with 5% CO_2_, the cell nuclei were stained and fixed using 1 mg/mL of 4′,6-diamidino-2-phenylindole (DAPI) dye in methanol. The stained cells were then incubated for 5 min in a dark room. Subsequently, the cells were examined using a fluorescence microscope (OLYMPUS, BX61, USA) (Saharkhiz et al., 2023a). To determine the relative cellular uptake of QDs-labeled Trz-pHSNs in SKBR-3 and L929 cell lines, 5× 10^5^ cells of each type were cultured in a 9-cm^2^ dish (Falcon 3001F). They were then treated with 250 μg/mL of QDs-dispersed Trz-pHSNs in the cell culture media and pure medium. Subsequently, they were incubated for 12 h at 37 °C with 5% CO^2^. The cells were washed four times with PBS and then collected from the dishes using a Costar cell scraper. The concentration of cells was measured using light scattering (Perkin-Elmer LS 5 spectrofluorometer, Waltham, USA). The cells were placed in a 3 × 3 mm^2^ fluorescence cuvette and then in the spectrofluorometer. Finally, the fluorescence intensity of each group was measured at 625 nm (Berg et al., 1990).

#### 2.4.10 Statistical analysis

Each set of data (*n* = 3) was reported as the mean and standard deviation (SD). The significance of the data was assessed using Prism software (version 9) with parametric analysis of variance (ANOVA) and Tukey's post hoc test. The outcomes were considered statistically significant at a *P* value of ≤0.001 for release tests and ≤ 0.05 for MTT assays.

## 3 Results and discussion

### 3.1 Characterization of CdSe/ZnS QDs

**Fig. 1:**
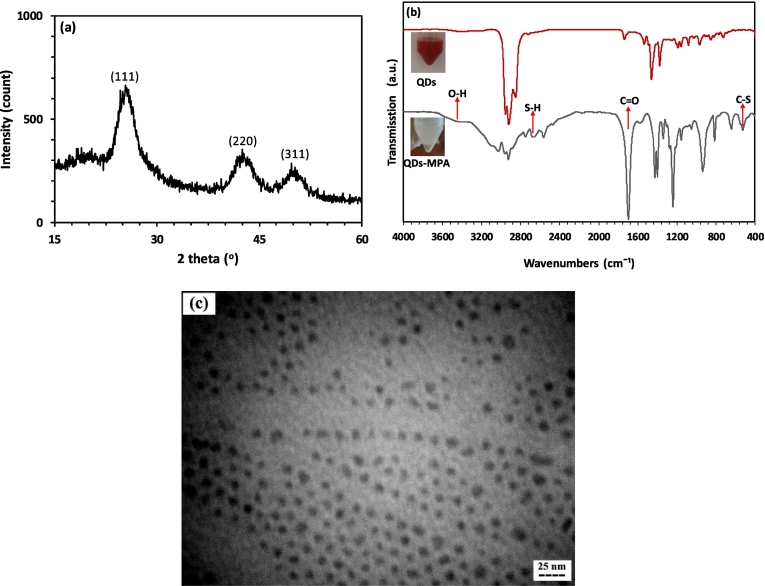
(a) XRD pattern of CdSe/ZnS core/shell QDs, (b) FTIR spectra of CdSe/ZnS QDs and MPA-capped CdSe/ZnS QDs, and (c) TEM image of CdSe/ZnS QDs.

Fig. 1a depicts the XRD pattern of the fabricated CdSe/ZnS core/shell QDs. The pattern reveals three distinct diffraction peaks at angles of 25.55, 42.4, and 50.6 degrees, corresponding to the (111), (220), and (311) diffraction planes, respectively, of the cubic zinc blend structure (ICDD PDF no. 00–019-0191). These findings provide evidence that CdSe successfully crystallized in the zinc blend structure (Rajan et al., 2017).

In this study, CdSe/ZnS nanocrystal QDs were synthesized using the nonpolar surfactant TOPO. However, to address the incompatibility and water dispersibility issues associated with these QDs, surface modification was essential. This was achieved by replacing TOPO with mercaptopropionic acid (MPA), which contains carboxyl and thiol (S—H) groups in its structure. The thiol group's strong electron affinity facilitated its interaction with the ZnS layer of QDs, enabling successful replacement. Moreover, the carboxyl group of MPA provided an excellent reactive site for immobilizing biological agents, such as antibodies (Rajan et al., 2017). To confirm the ligand exchange process, FTIR analysis was conducted. Fig. 1b displays the comparative FTIR spectra between CdSe/ZnS QDs and MPA-capped QDs. Three distinctive peaks in the FTIR spectrum of MPA-capped QDs, compared to CdSe/ZnS QDs, indicate successful modification. A prominent absorption band at ∼1697 cm^−1^ corresponds to the C=O stretching bond of carboxyl groups in MPA, confirming the replacement of TOPO with MPA. The appearance of a clear vibration C—S bond peak at ∼525 cm^−1^ after the ligand exchange process indicates that the ligand exchange of MPA with TOPO occurred successfully. Additionally, it seems that the carboxyl groups of MPA attached to the ZnS layer of QDs form a C—S bond. Moreover, an S—H bond is observed at ∼2661 cm^−1^ in the spectrum of MPA-capped QDs, while it is absent in the TOPO-capped ones, showing that some of the carboxyl groups on the surface of the QDs are still free. Furthermore, an O—H bond emerges at ∼3441 cm^−1^, facilitating the dispersion of MPA-capped QDs in water and biological media (Vo et al., 2015).

To assess the size and surface charge of the QDs, DLS and zeta potential measurements were conducted. Notably, the difference in size between the QDs and MPA-conjugated QDs was negligible, with both exhibiting a size of approximately 7 nm. However, after the replacement of TOPO with MPA, there was a significant shift in the surface charge of the QDs, transitioning from a positive charge of ∼ +15 mV to a negative charge of ∼ −10 mV. This shift indicated the transformation from the positively charged TOPO layer to the negatively charged MPA layer, validating the successful ligand exchange process (Vo et al., 2015; Vo et al., 2016). This observation aligned with the FTIR results, further confirming the effectiveness of the ligand exchange. Additionally, the TEM image of the QDs revealed uniform, homogeneous, and spherical particles with a diameter of about 5 nm (Fig. 1c). Importantly, no anomalously coarse particles were observed.

**Fig. 2:**
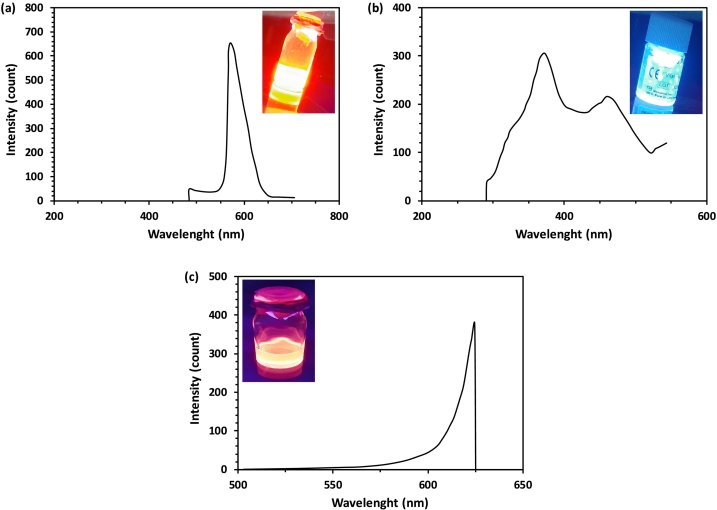
(a-c) Fluorescence spectra of TOPO-capped CdSe/ZnS QDs, MPA-capped CdSe/ZnS QDs, and Trz attached CdSe/ZnS QDs, respectively.

The attachment of CdSe/ZnS QDs to monoclonal antibodies is pivotal in biomedical applications such as bio-marking, bio-sensing, and bio-imaging (Fatima et al., 2021; Tiwari et al., 2009). The photoluminescence (PL) properties of the attached QDs are crucial for these applications. Therefore, to assess the PL changes during the conjugation process, fluorescence spectroscopy was performed on TOPO-capped CdSe/ZnS QDs, MPA-capped CdSe/ZnS QDs, and Trz antibody-conjugated CdSe/ZnS QDs (Trz-QDs). Based on the obtained spectra (Fig. 2), the emission wavelength of TOPO-capped QDs was ∼570 nm, representing orange light (Fig. 2a). After the replacement of TOPO ligands with MPA, the emission wavelength of the QDs shifted to ∼370 nm, corresponding to the blue color region (Fig. 2b). Additionally, the attachment of Trz to the QDs induced a significant red-shift in the emission wavelength, reaching ∼625 nm with a red color (Fig. 2c). These changes serve as evidence of the success of the conjugation process, confirming the observed results of DLS and zeta potential analyses (Shin et al., 2020). Similar photoluminescence changes during the conjugation process of QDs to protein molecules have been reported previously (Lv et al., 2021; Li et al., 2022).

### 3.2 Characterization of niosomal particles

**Fig. 3:**
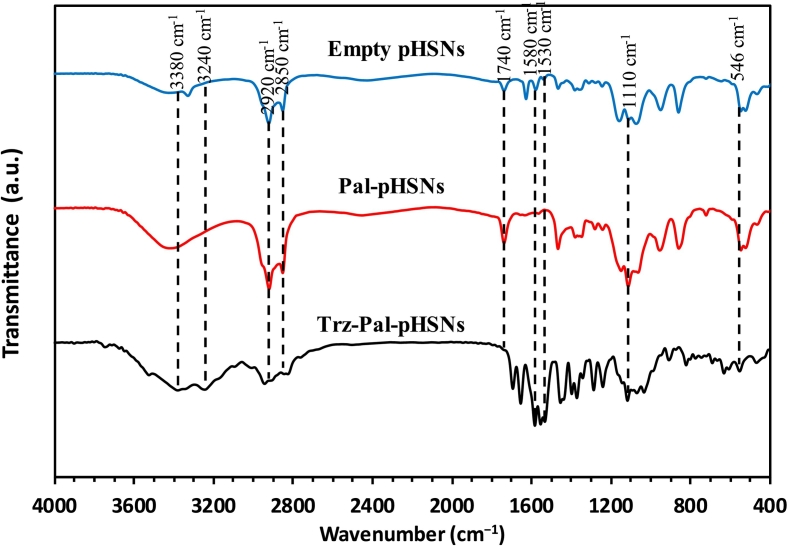
FTIR spectra of Empty-pHSN, Pal-pHSN, and Trz-Pal-pHSN.

Fig. 3 illustrates the FTIR spectra of Empty-pHSN, Pal-pHSN, and Trz-Pal-pHSN samples. The results reveal four distinct bands merged at ∼3380, ∼1110, ∼1740, and ∼ 2920 cm^−1^, attributed to the stretching O—H bond of the hydroxyl group, C—O stretching ester bond, C=O stretching ether bond, and C—H stretching bond of alkyl groups, respectively. These components are present in Span 60, Tween 60, and cholesterol components of pHSNs (Hua et al., 2006). Additionally, an S—H bond of thiol groups from the DSPE-PEG-Maleimide components emerged at ∼2850 cm^−1^ in all samples. However, this band exhibits lower intensity in the Trz-Pal-pHSNs compared to the others, indicating the incorporation of thiol groups into the formed disulfide bond between antibody molecules and maleimide groups of DSPE-PEG-Mal components. Furthermore, the unique band of the mentioned disulfide (S—S) bonds is observed at ∼546 cm^−1^ in the FTIR spectrum of Trz-Pal-pHSNs, confirming the successful attachment of Trz molecules to the maleimide groups (Torchynska et al., 2014). The presence of Trz molecules on the surface of the pHSNs is also supported by three peaks of stretching vibration of N—C (amide I), N=C (amide II), and binding vibration of N—H bonds at ∼1580, ∼1530, and ∼ 3240 cm^−1^, respectively, in the spectrum of Trz-Pal-pHSN. These three bonds correspond to the formed amide bond between MPA-QDs and Trz and residues of the protein structure of Trz (Nyquist, 2001; Rahimzadeh et al., 2021). The observation of the N—C bond in the unconjugated particles to the antibody is attributed to the pH-sensitive DSPE-CA-PEG2000 polymers in the structure of the pHSNs (Saharkhiz et al., 2023d; Ramírez-García et al., 2018).

To examine the alterations in size and surface charge of pHSNs before and after bio-conjugation with Trz, DLS, and zeta potential analyses were conducted. The Empty-pHSNs exhibited an average size of 41.9 ± 0.1 nm with a polydispersity index (PDI) of 0.017 ± 0.002, indicative of a homogeneous pHSN population. The encapsulation of Pal between the bilayers of the particles did not markedly affect the size of the pHSNs, revealing a size of 53.82 ± 1.04 nm with a PDI of 0.11 ± 0.005. However, the attachment of Trz monoclonal antibody molecules on the surface of the pHSNs significantly increased both their size and PDI to 169.2 ± 3.4 nm and 0.20 ± 0.02, respectively. Notably, the size within the range of 100 to 200 nm is favorable for the biodistribution of pHSNs, leveraging the enhanced permeability and retention (EPR) effect in cancer tissues (Zarrabi et al., 2021). Furthermore, the surface charge of Empty-pHSNs, Pal-pHSNs, and Trz-Pal-pHSNs were measured as −1.20 ± 0.01, −1.8 ± 0.1, and − 12.22 ± 0.03 mV, respectively. Prior studies have attributed the neutral surface charge of Empty-pHSNs and Pal-pHSNs to the presence of pH-sensitive DSPE-CA-PEG2000 components in their structure (Hua et al., 2006; Ramírez-García et al., 2018). Additionally, the negative zeta potential value for Trz-Pal-pHSNs confirms the bio-conjugation of the antibodies to the pHSNs (Maeda, 2012).

**Fig. 4:**
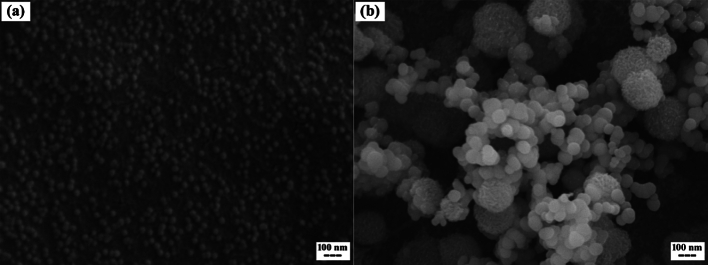
SEM images of (a) Pal-pHSNs, and (b) Trz-Pal-pHSNs.

Finally, to examine the morphological changes of pHSNs before and after surface modification with Trz, SEM analysis was conducted. The images revealed that Pal-pHSNs exhibited a homogeneous population of spherical particles with an average size of approximately 40 nm (Fig. 4a), consistent with the findings from the previously performed DLS analysis. Upon conjugation to Trz molecules, the size of the particles increased to ∼150 nm while maintaining a round-shaped appearance (Fig. 4b).

### 3.3 Bio-activity assessments

#### 3.3.1 Entrapment efficiency and loading capacity of Pal

As mentioned, Pal is a chemotherapeutic agent with poor solubility that impedes cell cycle progression from G1 to the S phase by inhibiting the phosphorylation of the Retinoblastoma (Rb) protein (Lee et al., 2019). The encapsulation of Pal molecules between the bilayers of pHSNs not only enhances their solubility but also reduces their side effects on normal cells. To assess the capacity of the fabricated pHSNs to load Pal molecules within their structure, the EE% and LC% of Pal were measured, yielding a value of 86.98 ± 3.1% and 31.93 ± 1.03, respectively. These values are comparable to the EE% and LC% of Pal in other previously optimized liposomal formulations conducted by Kommineni et al. and Li et al. (Gupta et al., 2019; Li et al., 2023).

#### 3.3.2 Number of herceptin attached to pHSNPs

Molecules to each pHSN involved performing the Bradford protein assay, followed by Brattlet's phosphorous assay. The Bradford assay results indicated that 4.432 × 10^4^ g of Trz molecules were attached to 1 mL of the final pHSN suspension. Simultaneously, there were 2.19 × 10^5^ mol of phosphorous in 1 mL of the mentioned suspension. Consequently, approximately 18 Trz molecules were attached to each pHSN particle, as calculated through Brattlet's phosphorous assay.

#### 3.3.3 Drug release pattern

**Fig. 5:**
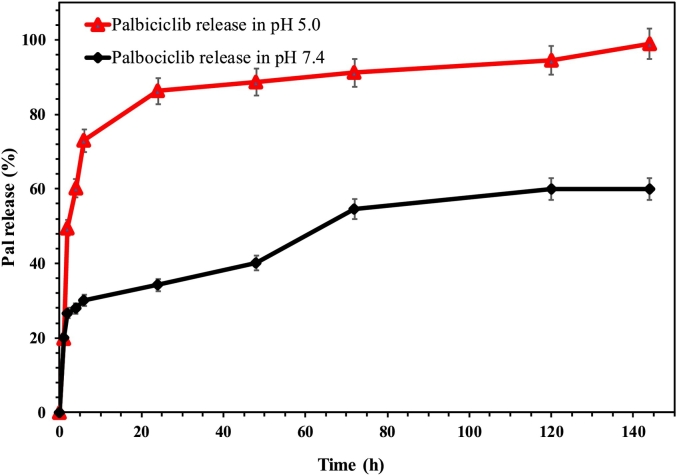
Pal release pattern from pHSNs at two pHs of 5.0 and 7.4 at 37 °C.

The release behavior of the fabricated Pal-pHSNs was assessed in two different pH mediums, 5.0 and 7.4, to simulate the acidic pH of lysosomes and normal tissues. As illustrated in Fig. 5, the release of Pal from the pHSNs at pH 5.0 exhibited two phases. In the initial phase (first two hours), an explosive release occurred due to the disruption of the Citraconic-amide bond in the DSPE-CA-PEG components, resulting in the separation of the CA-PEG layer. This structural change led to the release of entrapped drugs within the polymeric shell of the pHSNs (Ramírez-García et al., 2018; Kommineni et al., 2022). Subsequently, the release of encapsulated Pal within the pHSNs occurred in a more controlled manner due to the slight pH-sensitive nature of DSPE (Hua et al., 2006). Furthermore, the release pattern of the drug from pHSNs demonstrated a sustained release at pH 7.4. The final amount of released drug was approximately twice as much at pH 5.0 compared to pH 7.4, reaching nearly 100% release over 144 h, whereas this value was approximately 50% at pH 7.4. These findings confirm the pH-sensitivity of the prepared pHSNs, consistent with previously reported works, albeit with a higher amount of released drug (Hua et al., 2006; Ramírez-García et al., 2018; Cao et al., 2014). Additionally, the calculations demonstrated that the release of Pal from the pHSNs fitted to Higuchi model (Table 2) (Zhang et al., 2010; Saharkhiz et al., 2023c).

**Table 2:** R^2^ values of Pal release in different kinetic release models.

Model of kinetic release	R^2^ value pH 5 pH 7.4
Zero	0.9264 0.9021
First	0.9723 0.9109
Higuchi	0.9913 0.9906
Hixson-Crowell	0.8114 0.9221

#### 3.3.4 Stability of pHSNs

**Fig. 6:**
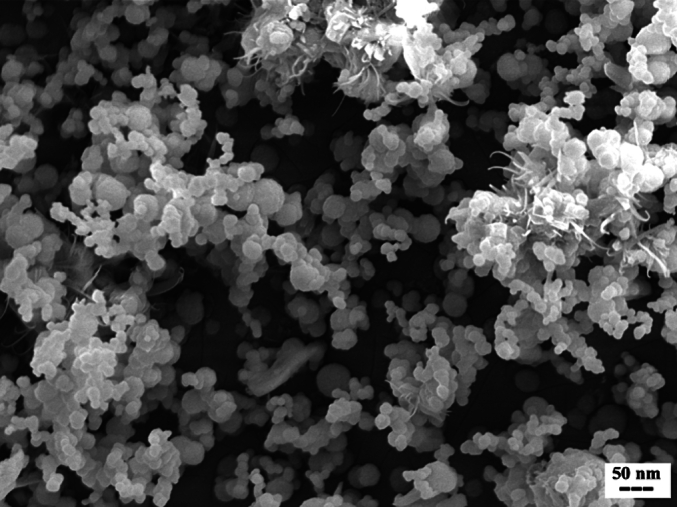
SEM image of Trz-Pal-pHSNs after 4 months of storage at 4 °C.

After storing the Trz-Pal-pHSNs suspension at 4 °C for four months, a reassessment of stability was conducted through SEM and DLS analyses, as well as EE% measurements. The size of the Trz-Pal-pHSNs was determined to be 183.2 ± 1.6 nm with a PDI of 0.25 ± 0.02. Additionally, the EE% of Pal was found to be 74.9 ± 0.5, indicating a negligible 12% drug leakage over the four months. The SEM image confirmed the DLS results, revealing no significant particle aggregation and an average size of 200 nm (Fig. 6). These findings suggest that the fabricated nanocarriers maintained sufficient stability over the observed period. Furthermore, evaluating their stability in different environmental conditions such as different pHs over the storage time is recommended.

#### 3.3.5 Integrity of red blood cell (RBC) count in the presence of Trz-pHSNs

**Fig. 7:**
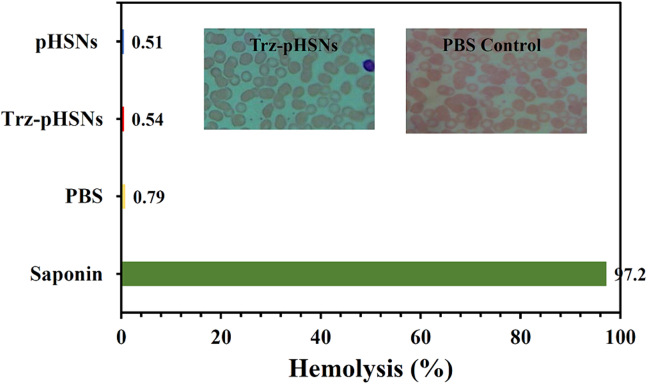
The hemolysis percentage of hemoglobin after contact with different samples.

According to the microscopical images no morphological change occurred in RBC after contact with the Trz-pHSNs. In addition, the hemolysis percentage (Fig. 7) of the RBCs after exposure to the saponin, PBS, pHSNs, and Trz-pHSNs was 97.2%, 0.79%, 0.51%, and 0.54%, respectively. Therefore, based on the previously reported literature, the Trz-pHSNs can be considered non-hemolytic (Moravej and Mantovani, 2011; Kuznetsova et al., 2012). It is noticeable that the prepared sample did not affect the cell count of the RBC and platelets (Table 3).

**Table 3:** The effect of prepared niosomal particles in hemocompatibility tests.

Niosomal formulation	RBC (10^6^ per μL, n = 3)	Platelet count (10^6^ per μL, n = 3)	Clotting time *a*PTT/PT	Hemolysis
Control solution	4.72 ± 0.11	0.415 ± 0.017	–	–
pHSNs	4.63 ± 0.18	0.410 ± 0.009	++/+	–
Trz-pHSNs	4.54 ± 0.21	0.400 ± 0.011	+/+	–

Note: –: weak or no effect, +: moderate effect, and ++: strong interference.

#### 3.3.6 Effect of Trz-pHSNs on coagulation

Activated partial thromboplastin time (*a*PTT) and prothrombin time (PT) are two common tests to evaluate the performance of the coagulation system. *a*PTT is a value to investigate the intrinsic activation pathway and PT is for assessment of the extrinsic one. Firstly, Plasma samples were recalcified to reverse the influence of an anticoagulant which is utilized during the blood collecting and supplying process (for the intrinsic pathway and the extrinsic pathway initiation, Cephaline plus kaolin suspension and human placental thromboplastin were used). Next, the measurement of clotting time was done. The clotting ability of the standard plasma was considered 100%. The clotting ability of each sample is lower than the standard plasma if it takes more time to clot in comparison to the standard plasma (Kuznetsova et al., 2012; Gorbet and Sefton, 2004). Table 3 presents the obtained results from the assay. According to them, *a*PTT was noticeably increased in the presence of the pHSNs and Trz-pHSNs. pHSNs and Trz-pHSNs caused approximately 16% and 44% of reduction in plasma clotting ability were observed at 0.45 mM and 0.045 mM final concentration in blood, respectively. These clotting ability reductions after exposure to pHSNs and Trz-pHSNs in the PT test were less than *a*PTT, demonstrating 8% and 21% reduction, respectively.

#### 3.3.7 Cytotoxicity induction

**Fig. 8:**
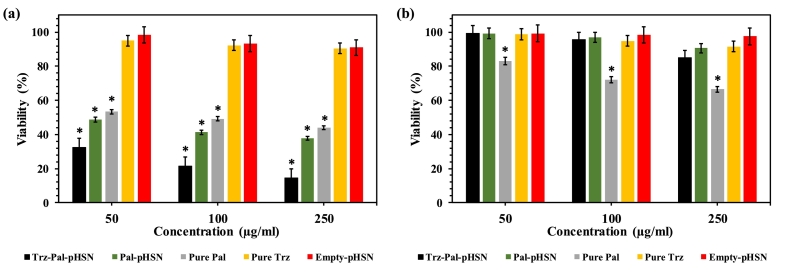
MTT assay results of (a) SKBR-3 cells, and (b) L929 cells after 24 h of incubation with different treatments at 37 °C. * is an indicator of the significance of the viability of cells after incubation with each treatment compared to the control group (*P* ≤ 0.05).

The cytotoxicity assessment of Pal encapsulated in pHSNs and targeted Trz-pHSNs was conducted, comparing with free Pal at equivalent concentrations on both SKBr-3 and L929 cell lines using the MTT assay after 24 h of incubation at 37 °C (Fig. 7). The cytotoxicity of the fabricated nanosystems exhibited a dose-dependent trend. As depicted, the viability of SKBr-3 cells decreased from 32.6% to 14.7% with an increase in the concentration of Trz-Pal-pHSNs from 50 to 250 μg/mL (Fig. 8a). Notably, the targeting effect with Trz was pronounced, with the cell death percentage caused by Pal-pHSNs being twice lower than that observed with Trz-targeted ones at all three concentrations (Fig. 8a) (Raju et al., 2013). The cytotoxicity performance of Pal encapsulated in both targeted and untargeted pHSNs surpassed that of pure Pal. It is hypothesized that upon encountering the external environment of cancer cells, the acidic pH conditions lead to a charge reversal from negative to positive in the DSPE-CA-PEG2000 components. This protonation of the components with water molecules causes the breakage of the amide bond between CA and DSPE, leading to the separation of the CA-PEG layer from the particle surface (Kommineni et al., 2022). The remaining positive charge on the pHSNs' surface facilitates attraction between the particles and the cell membrane. Subsequently, upon proximity to the cell membrane, Trz molecules specifically attach to the HER-2 receptors on the cell surface, activating endocytosis in HER-2 overexpressed cells (Hua et al., 2006; Ramírez-García et al., 2018; Lee et al., 2019). Additionally, the combination of Pal and Trz appears to induce a synergistic therapeutic effect on HER2-positive cancer cells (Namboodiri and Pandey, 2011). The biocompatibility of the fabricated treatments (i.e., Pal-pHSN, Trz-Pal-pHSN, and Empty-pHSN) was evaluated on normal L929 cells (Fig. 8b), revealing no significant cytotoxicity. Remarkably, the empty-pHSNs did not induce substantial cell death in SKBR-3 cells (∼91% viability at the concentration of 250 μg/mL), affirming the biocompatibility of the fabricated nanocarriers. To the best of our knowledge, this study is the first report of a Trz conjugated smart niosomal formulation for delivery of Pal. Previously, a phase II trial study was conducted by Ciruelos and coworkers which showed the combination of Pal and Trz showed a synergic effect in metastatic BC patients (Ciruelos et al., 2020; Escrivá-de-Romaní et al., 2018). However, Raikwar et al. (2023) developed a Trz-conjugated pH-sensitive liposome formulation for the delivery of Paclitaxel to the HER2-positive BC cells which inhibited the growth of SKBR-3 cells with a close rate to our report.

#### 3.3.8 Cellular apoptosis induction

**Fig. 9:**
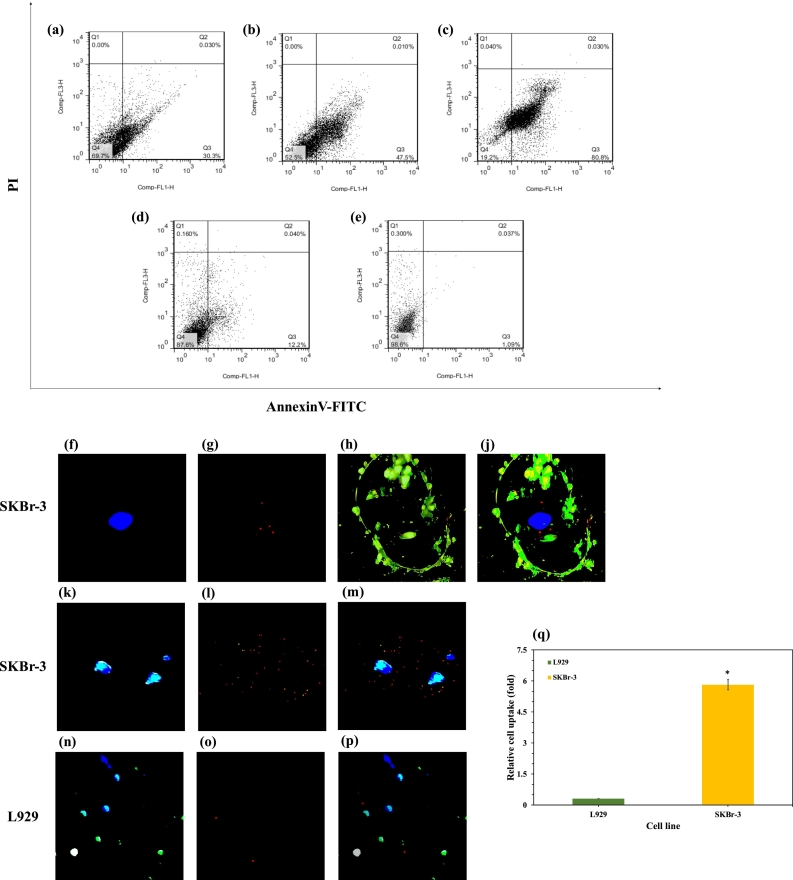
Flow cytometry apoptosis graphs of SKBR-3 cells after 24 h of incubation with (a) pure Pal, (b) Pal-pHSNs, (c) Trz-Pal-pHSNs, (d) Empty-pHSNs, and (e) control at 37 °C. DAPI-stained nucleus of (f) SKBR-3 cell, (g) CdSe/ZnS QDs, (h) Annexin V-FITC stained membrane of SKBR-3 cell, and (j) merged image of f, g, and h, (k) DAPI-stained nuclei of SKBR-3, (l) CdSe/ZnS QDs, (m) merged image of k and m, (n) DAPI-stained of L929 nuclei, (o) Cd-Se/ZnS QDs, (p) merged image of o and p, (q) relative internalization of Trz-pHSNs to the SKBr-3 cell line in comparison to the L929 cells (*P ≤ 0.05).

To assess the apoptotic response of different treatments to the HER2-positive SKBR-3 cell line after 2 h of incubation at 37 °C, a flow cytometry assay using a PI/Annexin V-FITC apoptosis kit was conducted. The results revealed that pure Pal induced approximately 30.33% total apoptosis in SKBR-3 cells. In contrast, incubation of cells with Pal encapsulated in pHSNs increased the total apoptosis rate to around 48% (Fig. 9a and b). This heightened rate of cell apoptosis induction by Pal-pHSNs compared to pure Pal is attributed to the formulation's ability to enhance the presence of drug molecules inside the cells (Hua et al., 2006). Furthermore, upon active targeting of Pal-pHSNs with Trz monoclonal antibodies, the total apoptosis rate of SKBR-3 cells significantly rose to approximately 81% (Fig. 9c). This outcome validates the specific endocytosis of the particles due to the lock-and-key interaction between Trz molecules and overexpressed HER-2 receptors on the cell surface (Ademuyiwa et al., 2023b). Additionally, apoptosis induced by Empty-pHSNs is negligible, underscoring the biocompatibility of the fabricated particles (Fig. 9d). These findings are consistent with the MTT results, providing further confirmation.

#### 3.3.9 Intracellular uptake study

CdSe/ZnS QDs possess numerous advantageous optical features compared to organic dyes, making them excellent bio-imaging agents. In this study, they were utilized to monitor the Trz-guided internalization of pHSNs to the HER2-positive cells (Vu and Claret, 2012). SKBR-3 cells, characterized by HER2 overexpression, and also L929 without expression of HER2 receptors were incubated with CdSe/ZnS QD-attached Trz-pHSNs for 24 h at 37 °C, followed by washing and double staining with DAPI and FITC-Annexin V dyes (Maxwell et al., 2020). The setup was examined under a fluorescence microscope, where Fig. 9f, and k display the DAPI-stained nucleus of the SKBR-3 cell, Fig. 9g, and l illustrates the CdSe/ZnS QDs emitting red light, and Fig. 9h shows the FITC-Annexin V-stained cell membrane. Upon merging the images in Fig. 9j, and m, the internalization and accumulation of Trz-QDs around the nucleus are evident, confirming the cellular uptake of Trz-pHSNs by HER2-positive cells (Miao et al., 2013). On the other hand, the obtained images after incubation of L929 cells with Trz-pHSNs demonstrated no noticeable accumulation of QDs around their nuclei, Fig. 9n to p. In addition, Fig. 9q represents the relative internalization of the Trz-pHSNs to the SKBr-3 cells in comparison to the L929, confirming that the fabricated Trz-pHSNs internalized to the SKBr-3 with approximately 21.9 fold more rate than their internalization rate to the L929 cells, due to the presence of HER2 receptors on the surface of SKBr-3 cell membrane (Wen et al., 2017).

Overall, this theranostic system, employing QD-attached Trz-pHSNs, can serve as a targeted probe for the specific diagnosis of HER2-positive BC cells, complementing its role in specific chemotherapeutic drug delivery. Despite these benefits of QDs for biomedical applications including tunable size, narrow spectra, high quantum yield, high sensitivity, etc.; there are still numerous notable drawbacks hindering the extensive utilization of QDs as bioimaging tools. QDs, unlike traditional organic fluorophores, act as nano colloids, which makes their prolonged application in biological settings complex and raises further safety issues. Some specific obstacles include limited solubility in water, intricate surface chemistry, lack of biological specificity, unregulated distribution to specific tissues, and the risk of severe long-term toxicity (McHugh et al., 2018; Kumar, 2016). QDs produced through conventional organic methods often exhibit hydrophobic surface characteristics, which can hinder their solubility and stability in aqueous environments for in vivo applications. Consequently, post-synthesis modifications such as encapsulation, ligand exchange, bioconjugation, or alternative approaches are necessary to enhance their surface properties (Mukherjee et al., 2016). Additional important limitations include the ability to only control the distribution of quantum dots (QD) to specific organs, reduce their long-term accumulation in the body, and limit their long-term toxicity (Hardman, 2006). The fate of these nanocolloids in vivo is ultimately determined by the type of QD chemistry and resulting physicochemical properties. Upon administration to the circulatory system, the particles face multiple defense mechanisms and must overcome significant biological barriers at both the organ and cellular levels. The absorption, distribution, metabolism, excretion, and toxicity of QDs depend on various properties, which need to be thoroughly studied before their clinical application (Hardman, 2006). To enhance the interaction of QDs with target cells, their composition and properties, such as particle size, surface charge, and hydrophilic coating, can be deliberately designed. This design can slow down the body's natural clearance mechanisms and improve the interaction with target cells (Bilan et al., 2016; Derfus et al., 2004). Surface modification with biological entities that specifically target receptors (such as Trz) has shown significant success in improving the aqueous solubility and cell/tissue specificity of imaging and diagnostic techniques. However, despite numerous published studies, there is still no widespread consensus on all the factors that influence the biodistribution of particles and their interactions with target cells (Derfus et al., 2004; Wang et al., 2012).

## 4 Conclusion

In this study, a novel theranostic approach was developed to address the limitations associated with the conventional use of chemotherapeutic drugs like Pal, which often exhibit side effects on normal cells. The key innovation involved the formulation of a targeted niosomal system with pH sensitivity, tailored for the acidic microenvironment of cancer tissues. Comprehensive analyses were conducted on the Trz-Pal-pHSNs, showcasing their potential for medical applications. The results indicated the successful preparation of these nanosystems, demonstrating suitable size and key characteristics. Bioactivity assessments affirmed the high specificity and affinity of the particles toward target cells. Moreover, the particles exhibited promising potential for antibody-guided bio-imaging. Overall, the findings suggest that the proposed approach holds promise for future advancements in cancer treatment and diagnosis.

## Author contributions

S Saharkhiz, N Nasri, and G Dini contributed to the conception and design of the study. S Saharkhiz, N Nasri, N Naderi, F Firoozbakht, and SS Ghalehshahi conducted formal analyses and investigations. S Saharkhiz and N Nasri performed the statistical analysis. S Saharkhiz and N Nasri wrote the first draft of the manuscript. G Dini as project administration provided the resources and validations. All authors contributed to the manuscript revision, read, and approved the submitted version.

## Funding

The authors declare that no funds, grants, or other support were received during the preparation of this manuscript.

## CRediT authorship contribution statement

**Shaghayegh Saharkhiz:** Writing – original draft, Visualization, Validation, Methodology, Investigation, Formal analysis, Conceptualization. **Negar Nasri:** Writing – original draft, Visualization, Methodology, Investigation, Formal analysis, Conceptualization. **Nazanin Naderi:** Investigation, Formal analysis. **Ghasem Dini:** Writing – review & editing, Validation, Supervision, Resources, Project administration, Conceptualization. **Saeid Shirzadi Ghalehshahi:** Investigation, Formal analysis. **Fateme Firoozbakht:** Formal analysis.

## Declaration of competing interest

The authors declare that the research was conducted in the absence of any commercial or financial relationships that could be construed as a potential conflict of interest.

## Data Availability

The datasets generated during and/or analyzed during the current study are available from the corresponding author upon reasonable request.

## References

[bb0005] Ademuyiwa F.O. (2023). A phase II study of palbociclib plus letrozole plus trastuzumab as neoadjuvant treatment for clinical stages II and III ER+ HER2+ breast cancer (PALTAN). Npj. Breast Cancer.

[bb0010] Ademuyiwa F.O. (2023). A phase II study of palbociclib plus letrozole plus trastuzumab as neoadjuvant treatment for clinical stages II and III ER+ HER2+ breast cancer (PALTAN). npj Breast Cancer.

[bb0015] Attia M.F. (2019). An overview of active and passive targeting strategies to improve the nanocarriers efficiency to tumour sites. J. Pharm. Pharmacol..

[bb0020] Berg K. (1990). Cellular uptake and relative efficiency in cell inactivation by photo activated sulfonated meso-tetraphenylporphines. Photochem. Photobiol..

[bb0025] Bilan R., Nabiev I., Sukhanova A. (2016). Quantum dot-based nanotools for bioimaging, diagnostics, and drug delivery. ChemBioChem.

[bb0030] Cao J. (2014). Polymeric micelles with citraconic amide as pH-sensitive bond in backbone for anticancer drug delivery. Int. J. Pharm..

[bb0035] Cheraghali S. (2023). PEG-coated MnZn ferrite nanoparticles with hierarchical structure as MRI contrast agent. Nanomaterials.

[bb0040] Chime S., Onunkwo G., Onyishi I. (2013). Kinetics and mechanisms of drug release from swellable and non swellable matrices: a review. Res. J. Pharm., Biol. Chem. Sci..

[bb0045] Choong G.M., Cullen G.D., O’Sullivan C.C. (2020). Evolving standards of care and new challenges in the management of HER2-positive breast cancer. CA Cancer J. Clin..

[bb0050] Ciruelos E. (2020). Palbociclib and trastuzumab in HER2-positive advanced breast cancer: results from the phase II SOLTI-1303 PATRICIA trial. Clin. Cancer Res..

[bb0055] Derfus A.M., Chan W.C., Bhatia S.N. (2004). Probing the cytotoxicity of semiconductor quantum dots. Nano Lett..

[bb0060] Di Francesco M. (2021). Doxorubicin hydrochloride-loaded nonionic surfactant vesicles to treat metastatic and non-metastatic breast cancer. ACS Omega.

[bb0065] Dvorakova V. (2017). An advanced conjugation strategy for the preparation of quantum dot-antibody immunoprobes. Anal. Methods.

[bb0070] Ekerljung L., Lindborg M., Gedda L., Frejd F.Y., Carlsson J., Lennartsson J. (2008). Dimeric HER2-specific affibody molecules inhibit proliferation of the SKBR-3 breast cancer cell line. Biochem. Biophys. Res. Commun..

[bb0075] Escrivá-de-Romaní S. (2018). HER2-positive breast cancer: current and new therapeutic strategies. Breast.

[bb0080] Faraji S., Dini G., Zahraei M. (2019). Polyethylene glycol-coated manganese-ferrite nanoparticles as contrast agents for magnetic resonance imaging. J. Magn. Magn. Mater..

[bb0085] Fatima I. (2021). Quantum dots: synthesis, antibody conjugation, and HER2-receptor targeting for breast cancer therapy. J. Funct. Biomater..

[bb0090] Gadag S. (2020). Combination therapy and nanoparticulate systems: smart approaches for the effective treatment of breast cancer. Pharmaceutics.

[bb0095] Gampenrieder S.P. (2020). Treatment landscape for patients with HER2-positive metastatic breast cancer: a review on emerging treatment options. Cancer Manag. Res..

[bb0100] Gharbavi M. (2018). Niosome: a promising nanocarrier for natural drug delivery through blood-brain barrier. Adv. Pharmacol. Sci..

[bb0105] Gorbet M.B., Sefton M.V. (2004). Biomaterial-associated thrombosis: roles of coagulation factors, complement, platelets and leukocytes. Biomaterials.

[bb0110] Gupta P., Narayanan S., Yang D.-H. (2019). Protein Kinase Inhibitors as Sensitizing Agents for Chemotherapy.

[bb0115] Hardman R. (2006). A toxicologic review of quantum dots: toxicity depends on physicochemical and environmental factors. Environ. Health Perspect..

[bb0120] Hua X.-F. (2006). Characterization of the coupling of quantum dots and immunoglobulin antibodies. Anal. Bioanal. Chem..

[bb0125] Kommineni N. (2022). Stealth liposomal chemotherapeutic agent for triple negative breast cancer with improved pharmacokinetics. Nanotheranostics.

[bb0130] Kumar S., Tan Mingqian, Wu Aiguo (2016). Nanomaterials for Tumor Targeting Theranostics. A Proactive Clinical Perspective.

[bb0135] Kuznetsova N.R. (2012). Hemocompatibility of liposomes loaded with lipophilic prodrugs of methotrexate and melphalan in the lipid bilayer. J. Control. Release.

[bb0140] Lee J.-Y., Shin D.H., Kim J.-S. (2019). Anticancer effect of metformin in herceptin-conjugated liposome for breast cancer. Pharmaceutics.

[bb0145] Li J. (2022). The role of surface charges in the blinking mechanisms and quantum-confined Stark effect of single colloidal quantum dots. Nano Res..

[bb0150] Li X. (2023). c-Myc-targeting PROTAC based on a TNA-DNA bivalent binder for combination therapy of triple-negative breast cancer. J. Am. Chem. Soc..

[bb0155] Lin A., Rugo H.S. (2007). The role of trastuzumab in early stage breast cancer: current data and treatment recommendations. Curr. Treat. Options in Oncol..

[bb0160] Lv Y. (2021). A CdSe/ZnS core/shell quantum dot-based fluorescence-linked immunosorbent assay for the sensitive and accurate detection of procalcitonin. Chem. Lett..

[bb0165] Maeda H. (2012). Macromolecular therapeutics in cancer treatment: the EPR effect and beyond. J. Control. Release.

[bb0170] Maxwell T. (2020). Nanoparticles for Biomedical Applications.

[bb0175] McHugh K.J. (2018). Biocompatible semiconductor quantum dots as cancer imaging agents. Adv. Mater..

[bb0180] Miao Q. (2013). Anticancer effects of bufalin on human hepatocellular carcinoma HepG2 cells: roles of apoptosis and autophagy. Int. J. Mol. Sci..

[bb0185] Moravej M., Mantovani D. (2011). Biodegradable metals for cardiovascular stent application: interests and new opportunities. Int. J. Mol. Sci..

[bb0190] Mukherjee A., Shim Y., Myong Song J. (2016). Quantum dot as probe for disease diagnosis and monitoring. Biotechnol. J..

[bb0195] Murthy R.K. (2014). Effect of adjuvant/neoadjuvant trastuzumab on clinical outcomes in patients with HER2-positive metastatic breast cancer. Cancer.

[bb0200] Namboodiri A., Pandey J. (2011). Differential inhibition of trastuzumab-and cetuximab-induced cytotoxicity of cancer cells by immunoglobulin G1 expressing different GM allotypes. Clin. Exp. Immunol..

[bb0205] Nyquist R.A. (2001).

[bb0210] Rahimzadeh Z. (2021). A rapid nanobiosensing platform based on herceptin-conjugated graphene for ultrasensitive detection of circulating tumor cells in early breast cancer. Nanotechnol. Rev..

[bb0215] Raikwar S. (2023). Antibody-conjugated pH-sensitive liposomes for HER-2 positive breast cancer: development, characterization, in vitro and in vivo assessment. J. Liposome Res..

[bb0220] Rajan M. (2017). Cytotoxicity assessment of palbociclib-loaded chitosan-polypropylene glycol nano vehicles for cancer chemotherapy. Mater. Today Chem..

[bb0225] Raju A., Muthu M.S., Feng S.-S. (2013). Trastuzumab-conjugated vitamin E TPGS liposomes for sustained and targeted delivery of docetaxel. Expert Opin. Drug Deliv..

[bb0230] Ramírez-García G. (2018). An immunoconjugated up-conversion nanocomplex for selective imaging and photodynamic therapy against HER2-positive breast cancer. Nanoscale.

[bb0235] Rana A. (2023). “Smart” drug delivery: a window to future of translational medicine. Frontiers. Chemistry.

[bb0240] Rocca A. (2014). Palbociclib (PD 0332991): targeting the cell cycle machinery in breast cancer. Expert. Opin. Pharmacother..

[bb0245] MA Sadat S. (2015). Nano-pharmaceutical formulations for targeted drug delivery against HER2 in breast cancer. Curr. Cancer Drug Targets.

[bb0250] Saharkhiz S., Zarepour A., Zarrabi A. (2023). A new theranostic pH-responsive niosome formulation for doxorubicin delivery and bio-imaging against breast cancer. Int. J. Pharm..

[bb0255] Saharkhiz S. (2023). Development of a new smart theranostic anti-PSMA-aptamer conjugated cationic-lipid coated mesoporous silica platform for targeted delivery of paclitaxel and CdSe/ZnS quantum dots to LNCaP cell line. J. Drug Deliv. Sci. Technol..

[bb0260] Saharkhiz S. (2023). A comparison study between doxorubicin and curcumin co-administration and co-loading in a smart niosomal formulation for MCF-7 breast cancer therapy. Eur. J. Pharm. Sci..

[bb0265] Saharkhiz S., Zarepour A., Zarrabi A. (2023). Empowering cancer therapy: comparing PEGylated and Non-PEGylated niosomes loaded with curcumin and doxorubicin on MCF-7 cell line. Bioengineering.

[bb0270] Shin D.H. (2016). Herceptin-conjugated temperature-sensitive immunoliposomes encapsulating gemcitabine for breast cancer. Arch. Pharm. Res..

[bb0275] Shin J.H., Shin D.H., Kim J.S. (2020). Let-7 miRNA and CDK4 siRNA co-encapsulated in Herceptin-conjugated liposome for breast cancer stem cells. Asian J. Pharm. Sci..

[bb0280] Tiwari D.K. (2009). Synthesis and characterization of anti-HER2 antibody conjugated CdSe/CdZnS quantum dots for fluorescence imaging of breast cancer cells. Sensors.

[bb0285] Torchynska T.V. (2014). The influence of bio-conjugation on photoluminescence of CdSe/ZnS quantum dots. Phys. B Condens. Matter.

[bb0290] Tsilidis K.K. (2023). Postdiagnosis body fatness, recreational physical activity, dietary factors and breast cancer prognosis: Global Cancer Update Programme (CUP Global) summary of evidence grading. Int. J. Cancer.

[bb0295] Vo N. (2015). Conjugation of *E. coli* O157: H7 antibody to CdSe/ZnS quantum dots. J. Nanomater..

[bb0300] Vo N.T. (2016). Stability investigation of ligand-exchanged CdSe/ZnS-Y (Y= 3-mercaptopropionic acid or mercaptosuccinic acid) through zeta potential measurements. J. Nanomater..

[bb0305] Vu T., Claret F.X. (2012). Trastuzumab: updated mechanisms of action and resistance in breast cancer. Front. Oncol..

[bb0310] Wang A.Z., Langer R., Farokhzad O.C. (2012). Nanoparticle delivery of cancer drugs. Annu. Rev. Med..

[bb0315] Wang, X., et al. n.d., Smart Drug Delivery Systems for Precise Cancer Therapy. (2211–3835 (Print)). 10.1016/j.apsb.2022.08.013 PMC9643298 36386470

[bb0320] Wen L. (2017). Tracking single baculovirus retrograde transportation in host cell via quantum dot-labeling of virus internal component. J. Nanobiotechnol..

[bb0325] Wu X. (2015). A fluorescence active gold nanorod–quantum dot core–satellite nanostructure for sub-attomolar tumor marker biosensing. RSC Adv..

[bb0330] Zarrabi A. (2021). Synthesis of curcumin loaded smart pH-responsive stealth liposome as a novel nanocarrier for cancer treatment. Fibers.

[bb0335] Zhang H. (2017). Liposomes: Methods and Protocols.

[bb0340] Zhang Y. (2010). DDSolver: an add-in program for modeling and comparison of drug dissolution profiles. AAPS J..

[bb0345] Zhen Z. (2015). Hemolysis and cytotoxicity mechanisms of biodegradable magnesium and its alloys. Mater. Sci. Eng. C.

